# Detecting and exploring kidney-derived extracellular vesicles in plasma

**DOI:** 10.1007/s10157-024-02464-z

**Published:** 2024-03-04

**Authors:** Shintaro Komatsu, Noritoshi Kato, Hiroki Kitai, Yoshio Funahashi, Yuhei Noda, Shoma Tsubota, Akihito Tanaka, Yuka Sato, Kayaho Maeda, Shoji Saito, Kazuhiro Furuhashi, Takuji Ishimoto, Tomoki Kosugi, Shoichi Maruyama, Kenji Kadomatsu

**Affiliations:** 1 Department of Nephrology, Nagoya University Graduate School of Medicine, Nagoya, Aichi Japan; 2 Division of Molecular Oncology, Center for Neurological Diseases and Cancer, Nagoya University Graduate School of Medicine, Nagoya, Aichi Japan; 3 Department of Biochemistry, Nagoya University Graduate School of Medicine, Nagoya, Aichi Japan

**Keywords:** Biomarker, Extracellular vesicles, Kidney, Plasma, Transplantation

## Abstract

**Background:**

Extracellular vesicles (EVs) have received considerable attention as ideal biomarkers for kidney diseases. Most reports have focused on urinary EVs, that are mainly derived from the cells in the urinary tract. However, the detection and the application of kidney-derived EVs in plasma remains uncertain.

**Methods:**

We examined the kidney-derived small EVs (sEVs) in plasma that were supposedly released from renal mesangial and glomerular endothelial cells, using clinical samples from healthy controls and patients with kidney transplants. Plasma from healthy controls underwent ultracentrifugation, followed by on-bead flow cytometry, targeting α8 integrin, an antigen-specific to mesangial cells. To confirm the presence of kidney-derived sEVs in peripheral blood, plasma from ABO-incompatible kidney transplant recipients was ultracentrifuged, followed by western blotting for donor blood type antigens.

**Results:**

Immunohistochemistry and immunoelectron microscopy confirmed α8 integrin expression in kidney mesangial cells and their sEVs. The CD9-α8 integrin double-positive sEVs were successfully detected using on-bead flow cytometry. Western blot analysis further revealed transplanted kidney-derived sEVs containing blood type B antigens in non-blood type B recipients, who had received kidneys from blood type B donors. Notably, a patient experiencing graft kidney loss exhibited diminished signals of sEVs containing donor blood type antigens.

**Conclusion:**

Our findings demonstrate the potential usefulness of kidney-derived sEVs in plasma in future research for kidney diseases.

**Supplementary Information:**

The online version contains supplementary material available at 10.1007/s10157-024-02464-z.

## Introduction

Renal biopsy is an important diagnostic technique providing nephrologists with information for managing patients with kidney diseases. Nonetheless, certain risks are associated with this procedure [1]. The establishment of a diagnostic method that does not require renal biopsy is thus desirable, leading to increased attention to non-invasive liquid biopsy [2].

Recently, studies have focused on extracellular vesicles (EVs) as potential biomarkers for non-invasive diagnosis [3, 4]. EVs contain mRNAs, miRNAs, and proteins derived from the cells from which they originate, and analyzing them may reveal the state of the cells of their origin. In kidney diseases, the majority of reports focus on urine samples as a source of EVs [5, 6]. Urine is an ideal biological sample to obtain information regarding cells located in the urinary tract, including the renal tubule, ureter, or bladder. There exists a subset of urinary EVs, enriched with aquaporin-2, a marker indicative of tubular cells [7]. However, for investigating endothelial or mesangial cells, which are separated from the urinary space by a tight basement membrane [8], plasma-derived EVs would be another source of examination.

Currently, it is not yet clear how EVs differ between the outer and inner spaces of the basement membrane, specifically between urinary EVs and plasma EVs. Studies analyzing the proteome of urinary EVs have shown that while most of the proteins present in these vesicles are expressed in the genitourinary system [9, 10], there is also evidence suggesting the existence of extrarenal sources [11]. In terms of disease biomarkers, most research on urinary EVs is associated with genitourinary diseases [12], and only few studies discuss their connection with other diseases [13, 14]. In our previous studies using in vitro systems, we showed that tubular-derived EVs cannot pass through the basement membrane under normal conditions [15]. Considering the tight barrier formed by the basement membrane, it can be inferred that there is a certain difference between the EVs of glomerular and those of the exterior lumen. Although plasma-derived EVs can be a mixture of EVs from various organs, they may offer an additional option for investigating kidney conditions related to mesangial and glomerular endothelial cells.

On-bead flow cytometry can detect EVs containing kidney-specific antigens among vesicles from diverse sources [4, 16, 17]. In this study, we detected the signals of plasma-derived EVs containing markers for mesangial cells. Then, we demonstrated the presence of kidney-derived EVs in peripheral blood using an ABO-incompatible kidney transplant. Our findings may contribute to future research on plasma-derived EV-based biomarkers for kidney diseases.

## Materials and methods

### Study participants

We enrolled seven kidney transplant recipients and 10 healthy volunteers from the Nagoya Kidney Disease Registry (NKDR). Kidney histology studies were conducted using biopsy specimens obtained from the NKDR. Informed consent was obtained from all individual participants included in the study. The study adhered to the Declaration of Helsinki (revised in Brazil, 2013) and was approved by Nagoya University’s Ethics Committee (approval number: 2010-1135-4).

### Plasma sample collection

From participants, 7 mL blood was collected in EDTA-coated tubes and centrifuged at 2000×*g* for 10 min at 4 °C. Supernatants were aliquoted and stored at − 80 °C until analysis.

### Cell culture

Normal human mesangial cells (NHMCs; CC-2559) were obtained from Lonza (Basel, Switzerland) and cultured in a supplemented mesangial basal medium (CC-3147, CC-4146) [18]. Human umbilical vein endothelial cells (HUVECs; C-12200) were sourced from PromoCell (Heidelberg, Germany) and grown in an endothelial cell growth medium kit (C-22110) [19]. Before EV isolation, cells were cultured in a medium containing EV-depleted FBS.

### Isolation of small EVs

To isolate small EVs (sEVs), plasma (0.4 mL) was centrifuged at 2000×*g* for 10 min, mixed with 11 mL of PBS, and then filtered through a 0.22 µm filter (FJ25BSCPS002AL01; GVS S.p.A., Bologna, Italy) To isolate sEVs for western blotting (WB) of α8 integrin (ITGA8), 5 mL of plasma was used. Cell culture supernatant (35 mL) containing EV-depleted FBS was also centrifuged at 2000×*g* for 10 min and subsequently filtered using the same 0.22 µm filter. The samples underwent ultracentrifugation at 175,000×*g* for 2 h using SW 32Ti rotor and Optima L-100Xp (Beckman Coulter, Brea, CA) [20, 21]. sEVs for immunoprecipitation had a pre-enrichment ultracentrifugation step before anti-CD63 antibody screening. For further analyses, the pellet was re-suspended in PBS, ultracentrifuged for washing, and then re-suspended for downstream procedures.

### Transmission electron microscopy

Beads or sEVs were placed on a carbon film-coated grid (645; Nisshin EM, Tokyo, Japan) and negatively stained with 2% uranyl acetate for 1 min. The samples were observed under a transmission electron microscope (JEM-1400 Plus; JEOL Ltd., Tokyo, Japan).

### Nanoparticle tracking analysis

Nanoparticle tracking was conducted using NanoSight NS300 (Malvern Panalytical, Malvern Worcestershire, UK). sEVs were diluted in 1000 µL of PBS with the following settings: camera level at 13, detection threshold at 5, and analysis temperature at 25 °C. Particle counts were converted to per mL of plasma.

### Protein quantification

Protein quantification used the Dot-it-Spot-it assay (Uppsala Biomedical Centre, Uppsala, Sweden). sEVs were re-suspended in Laemmli buffer (161-0737; Bio-Rad Laboratories, Hercules, CA). A diluted sample was applied to a nitrocellulose membrane, dried, and scanned. ImageJ (version 1.53) [22] analyzed the scans, measuring dot thickness. The Rodbard model determined sample concentrations using a standard curve.

### Western blot analysis

WB was performed for CD9, FLOT-1, and blood type B antigen. sEVs in Laemmli buffer were treated with 2-mercaptoethanol (21417-52; Nacalai Tesque, Kyoto, Japan) for FLOT-1 and blood type B antigen, then heated at 95 °C, separated using SDS-PAGE. and transferred onto Immobilon-P PVDF membranes (IPVH00010; Merck, Darmstadt, Germany). Blots were blocked, then incubated with primary antibodies: anti-CD9 (1:1000 dilution; MEX001-6, MBL, Tokyo, Japan), anti-CD63 (1:1000 dilution; MEX002-6, MBL), anti-FLOT-1 (1:5000 dilution; ab133497, Abcam, Cambridge, UK), anti-ITGA8, 1:1000 dilution, sc-365798, Santa Cruz Biotechnology, Dallas, TX), and anti-blood type B antigen (1:200 dilution; 448-03802, Fujifilm Wako, Osaka, Japan). Following washes, membranes were exposed to HRP-conjugated secondary antibodies: streptavidin (1:10,000 dilution; ab7403, Abcam), anti-rabbit IgG (1:4000 dilution; 111-035-144, Fujifilm), and anti-Mouse (1:20,000 dilution; 315-035-048; Jackson ImmunoResearch, West Grove, PA), in Can Get Signal Solution 2 (NKB-301; TOYOBO, Osaka, Japan). Blots were visualized with Immobilon Western Chemiluminescent HRP Substrate (WBKLS0500; Merck) or SuperSignal West Femto Maximum Sensitivity Substrate (34095; Thermo Fisher Scientific, Waltham, MA).

### Histology

Kidney samples were fixed in 10% formalin, embedded in paraffin, and sectioned at 1 µm. Following deparaffinization, immunohistochemistry and immunofluorescence were carried out as described previously [23]. For immunohistochemistry, sections were blocked using 10% normal goat serum (03953-66; Nacalai Tesque) and then stained with primary antibodies: anti-blood type A antigen (111006; Ortho, Raritan, NJ), anti-blood type B antigen (112003; Ortho), and anti-ITGA8 (NBP1-86519; Novus Biologicals). Secondary antibodies used were goat anti-mouse (424131; Nichirei Bioscience, Tokyo, Japan) and goat anti-rabbit (424141; Nichirei Bioscience), with visualization using 3,3′-diaminobenzidine (415171; Nichirei Bioscience). For immunofluorescence, sections were blocked using Blocking one histo (06349-64; Nacalai Tesque) and then stained with primary antibodies: anti-CD31 (ab28364; Abcam), anti-B (112003; Ortho), anti-Nephrin (GP-N2; PROGEN, Germany), anti-platelet-derived growth factor receptor Β (PDGFRB; sc-374573; Santa Cruz Biotechnology), and anti-ITGA8 (NBP1-86519; Novus Biologicals, Centennial, CO). Secondary antibodies applied were Alexa Fluor 555-conjugated goat anti-rabbit IgG (A21428; Invitrogen), Alexa Fluor Plus 647-conjugated donkey anti-mouse IgG (A32787; Invitrogen, Waltham, MA), Alexa Fluor 488-conjugated goat anti-guinea pig IgG (A-11073; Thermo Fisher Scientific), goat anti-mouse IgG, Alexa Fluor 488 (115-545-166; Jackson ImmunoResearch Laboratories, West Grove, PA). For immunofluorescence of ITGA8 in NHMCs and HUVECs, cells were fixed, then stained using mouse anti-human ITGA8 (sc-365798; Santa Cruz Biotechnology) and FITC-conjugated rabbit anti-mouse IgG (61-6511; Invitrogen). Images were obtained using BZ-X800 microscopy (Keyence, Osaka, Japan).

### Immunoelectron microscopy of sEVs from cultured NHMCs

sEVs from NHMC and HUVEC supernatants cultured in EV-depleted FBS were extracted under standard centrifugation conditions. Using Exosome-human CD63 Isolation/Detection Reagent (10606D; Thermo Fisher Scientific), the sEVs were captured with anti-CD63 antibody-coated beads. These were fixed in formalin for 10 min, then stained for ITGA8 (human integrin α8 antibody clone 481709: MAB6194; R&D Systems, Minneapolis, MN) or a control antibody. The secondary reaction utilized horseradish peroxidase-conjugated goat F(ab′)2 fragment anti-mouse IgG (Histofine, Nichirei Bioscience). Following 1% glutaraldehyde fixation and 3,3′-diaminobenzidine incubation, the samples underwent post-fixation in osmium tetroxide alcohol dehydration and were observed under an electron microscope (JEM-1400 Plus).

### Immunoprecipitation using anti-CD63 antibody with sEVs derived from human plasma

Antibody-conjugated magnetic beads were prepared using anti-CD63 antibody (MEX002-6; MBL), isotype control antibody (400304; BioLegend, San Diego, CA), and ExoCap streptavidin Kit (MEX-SA; MBL). Per the manufacturer’s instructions, 1000 µg of beads were coupled with 5 µg of biotinylated antibody at room temperature for 1 h, utilizing 50 µg of beads per reaction. Isolated sEVs (250 µL) combined with 250 µL treatment buffer were added to these beads and incubated at 4 °C overnight with rotation. After magnetically separating and washing thrice, the beads were re-suspended in a suitable buffer for further analysis.

### Immunoprecipitation using anti-blood type B antigen antibody with sEVs derived from human plasma

Five microliters of anti-blood type B antigen antibody (448-03802, Fujifilm Wako) or its isotype control antibody (14-4752-82; eBioscience, San Diego, CA) were added to the isolated sEVs. The samples were then incubated overnight at 4 °C, followed by the addition of protein L magnetic beads (88849; Thermo Fisher Scientific). After washing, the supernatants were removed using magnetic separation, and the sEVs captured by the antibody-conjugated beads were detached from the magnetic beads using glycine–HCl (pH 2.5) for 10 min. The sEVs in the supernatant were subsequently collected and neutralized by adding Tris–HCl (pH 9.0). The collected sEVs were used for downstream analysis.

### On-bead flow cytometry analysis of sEVs

Using 10 µg of the previously prepared 50 µg beads, on-bead flow cytometry was performed. Detection antibodies were prepared in 0.1% BSA/PBS: anti-CD9-FITC (1:200; MEX001-4, MBL), isotype control for CD9 mouse IgG2a-FITC (1:200; 076-4, MBL), anti-ITGA8-Alexa Fluor 488 (1:200; FAB6194G, R&D Systems), and its isotype control mouse IgG2b-Alexa Fluor 488 (1:66.7; IC004G, R&D Systems). After supernatant removal, the bead-bound sEVs were incubated with these antibodies at 4 °C for 30 min in darkness, with gentle agitation. Post-incubation, samples underwent two washes with PBS on a magnetic stand, then were re-suspended in 250 µL PBS for flow cytometry on a BD FACSCanto II (BD Biosciences, Franklin Lakes, NJ). Data was analyzed using FlowJo v10.8.1 (BD Biosciences), and the fluorescent signals were counted as mean fluorescent intensity (MFI).

### Statistical analysis

Statistical analysis was performed using Python 3.8.3 (Python Software Foundation, Wilmington, DE), and box plots were constructed using the “seaborn” module. Comparisons were evaluated using the Mann–Whitney *U* test, with statistical significance set at *p* < 0.05.

## Results

### Characterization of EVs isolated using ultracentrifugation

**Fig. 1:**
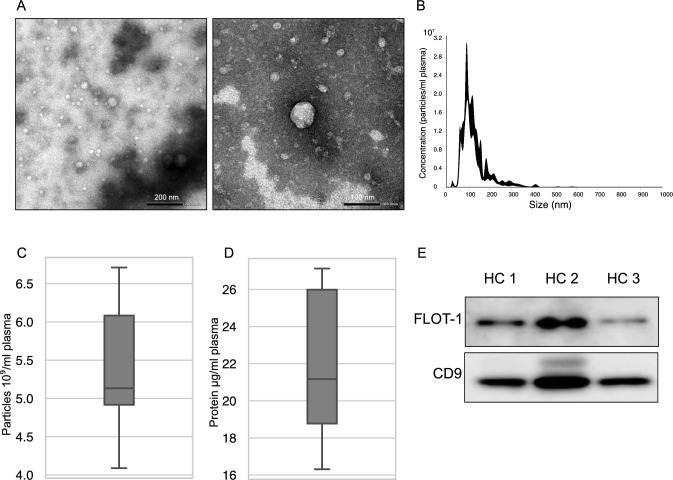
Characterization of plasma-derived sEVs. **A** Electron microscopy images of plasma-derived sEVs. **B** Representative image of the size distribution of the plasma-derived sEVs examined using nanoparticle tracking analysis. **C** The number of plasma-derived particles (*N* = 5). **D** Amount of protein extracted from the plasma using ultracentrifugation (*N* = 6). **E** WB analysis of plasma-derived sEVs using ultracentrifugation. FLOT-1 and CD9 are general sEV markers. *sEVs* small extracellular vesicles, *HC* healthy controls, *FLOT-1* flotillin-1

We isolated sEVs from human plasma using ultracentrifugation. In electron microscopy, the extracted particles were round or cup-shaped and satisfied the previously reported characteristics of sEVs (Fig. 1A) [15]. Nanoparticle tracking analysis revealed that the median concentration of isolated sEVs was 5.13 × 10^9^ particles per mL of plasma (Fig. 1B, C). In the protein assay, the median amount of protein in sEVs was 21.2 µg/mL plasma (Fig. 1D). WB analysis showed that the classical sEV markers, FLOT-1 and CD9, were positive in sEVs (Fig. 1E).

### α8 integrin (ITGA8) expressed in sEVs derived from renal mesangial cells

**Fig. 2:**
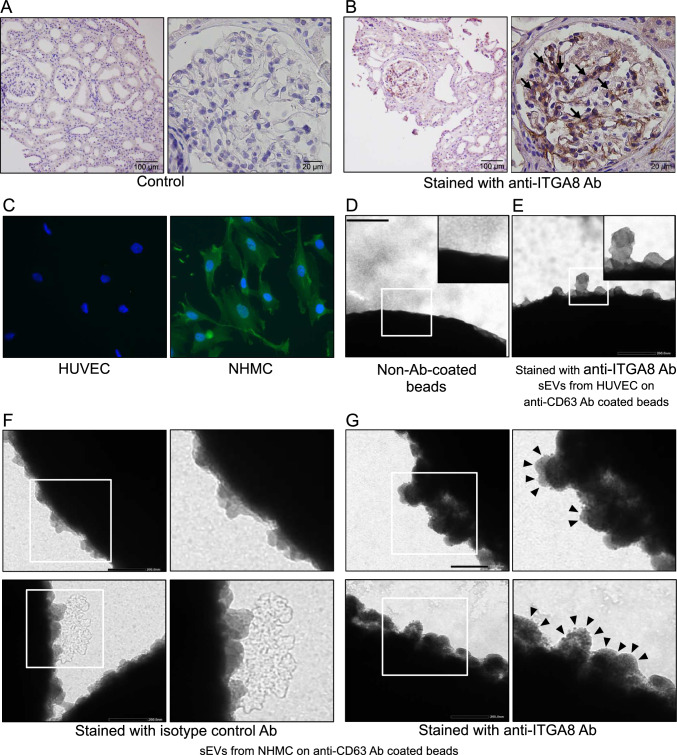
ITGA8 expression in mesangial cells in the biopsy specimen, NHMCs, and sEVs isolated from the NHMC supernatant. **A** Control immunohistochemistry images of human renal biopsy specimens without using primary antibody. Scale bars indicate 100 µm (left) and 20 µm (right). **B** The same specimen used in **A** stained with the anti-ITGA8 antibody. The arrows indicate positive staining in the glomerular mesangial area. Scale bars indicate 100 µm (left) and 20 µm (right). **C** Immunofluorescence images of HUVECs and NHMCs stained with DAPI (blue) and ITGA8 (green). **D**–**G** Immunoelectron microscopy images of sEVs capturing beads. Non-antibody-coated beads (**D**) and anti-CD63 (general sEV marker) antibody-coated beads with sEVs isolated from the supernatant of HUVECs (**E**) and NHMCs (**F**, **G**). Beads were then stained with either anti-ITGA8 antibody (**E**, **G**) or its isotype control antibody (**F**). The magnified images are shown on the right upper or the right side of the images. The arrowheads indicate small dots of positive staining for ITGA8. Scale bars indicate 200 nm. *sEVs* small extracellular vesicles, *NHMCs* normal human mesangial cells, *HUVECs* human umbilical vein endothelial cells, *ITGA8* α8 integrin, *Ab* antibody

Next, we examined whether we could detect kidney-derived sEVs expressing kidney-specific antigens in the peripheral blood using on-bead flow cytometry. Among the kidney cells, we focused on mesangial cells because they are located in the intravascular area that is not separated from the bloodstream by a basement membrane. We examined several markers for mesangial cells [24, 25], and then focused on ITGA8 as a mesangial cell marker [26, 27]. We confirmed ITGA8 expression in mesangial region in a human renal biopsy specimen using immunohistochemistry (Fig. 2A, B). In addition, we detected co-expression of ITGA8 with PDGFRB, which is primarily expressed in mesangial cells in the kidney, through immunofluorescence (Fig S1). Furthermore, ITGA8 was positively identified in a primary culture of NHMCs using immunofluorescence staining in vitro (Fig. 2C). We performed electron and immunoelectron microscopies on sEVs from the NHMC supernatant to evaluate ITGA8 expression in sEVs from mesangial cells. In immunoelectron microscopy, antibodies conjugated with HRP that bind to ITGA8 induced the precipitation of DAB (3,3′-diaminobenzidine), leading to a dense deposition on the surface of sEVs. Compared to that in non-antibody-conjugated beads (Fig. 2D), sEVs obtained from HUVECs were observed on anti-CD63 antibody-coated beads, a general marker for EVs, but without DAB deposition (Fig. 2E). In fact, such deposition was only observed on the surface of sEVs isolated from the NHMCs (Fig. 2F, G).

### Evaluation of ITGA8-positive sEVs in plasma

**Fig. 3:**
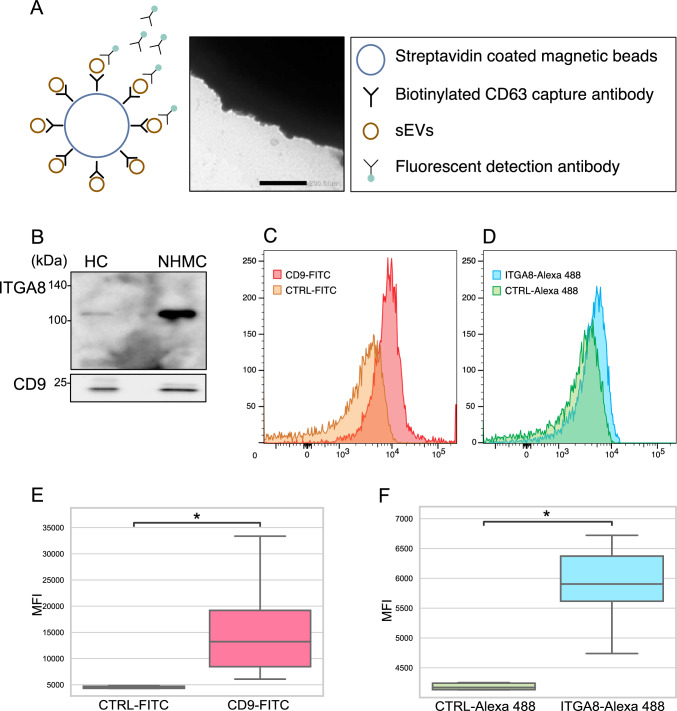
Detection of ITGA8-positive sEVs from human plasma samples. **A** Schematic diagram showing the ITGA8-positive sEVs detection system and an electron microscopy image of plasma-derived sEVs captured using anti-CD63 antibody-coated beads. Scale bar indicates 200 nm. **B** Western blotting analysis of a lysate from sEVs isolated from the plasma of the healthy control and a lysate from NHMC. **C**, **D** Representative images of sEVs signal detected using on-bead flow cytometry for CD9 (**C**) and ITGA8 (**D**). **E**, **F** Boxplots comparing MFI between anti-CD9 antibody-FITC and isotype control antibody-FITC (**E**) and between anti-ITGA8 antibody-Alexa flour and isotype control antibody-Alexa flour on anti-CD63 antibody-conjugated magnetic beads (**F**). *sEVs* small extracellular vesilces, *HC* healthy control, *NHMC* normal human mesangial cell, *ITGA8* α8 integrin, *MFI* mean fluorescent intensity, *CTRL* control. *p < 0.05

ITGA8-positive sEVs in human plasma were quantified using on-bead flow cytometry. CD63 antibody-conjugated magnetic beads were used to capture plasma sEVs. We visualized on-bead plasma-derived sEVs using electron microscopy (Fig. 3A). In addition, ITGA8 presence in sEVs isolated from the plasma of the healthy control was confirmed using WB (Fig. 3B). Following the reaction with the fluorescent antibody, we quantified the MFI of the on-bead sEVs. Representative flow cytometry images are shown in Fig. 3C and D. We performed on-bead flow cytometry on 10 healthy control (HC) plasma samples and successfully detected signals of CD63-CD9 and CD63-ITGA8 double-positive sEVs (Fig. 3E, F).

### Kidney-derived sEVs isolated from the plasma of kidney transplantation recipients

**Fig. 4:**
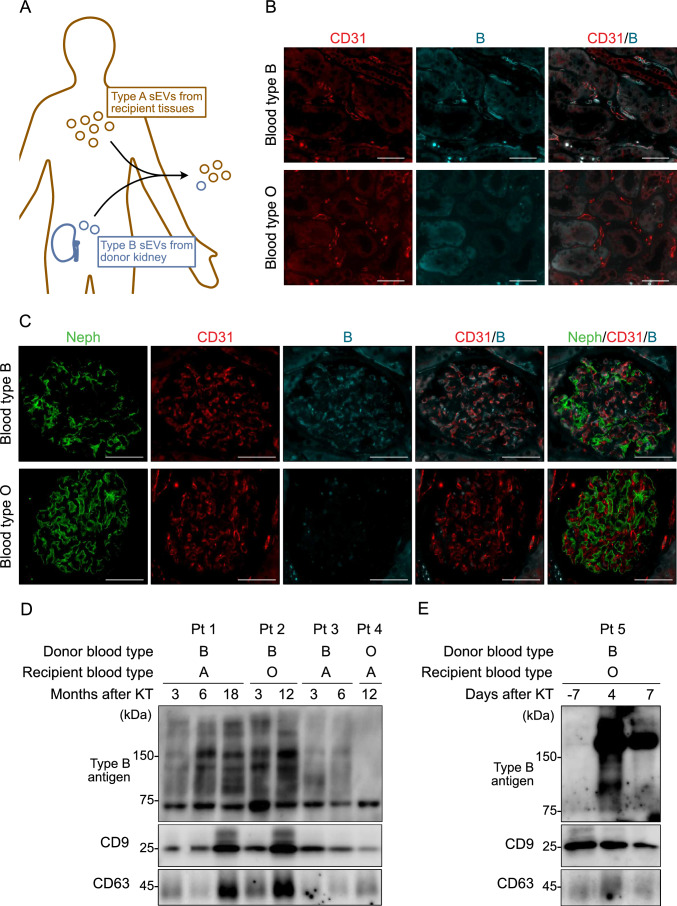
Detection of kidney-derived sEVs in the peripheral blood. **A** Schematic image showing the detection of kidney-derived sEVs from ABO-incompatible kidney transplant patients. **B**, **C** Immunofluorescent images of renal biopsy specimens from kidney transplant patients. **B** Peritubular capillary in kidney interstitium stained with anti-CD31 (red) and anti-blood type B antigen (blue). Scale bars indicate 50 µm. **C** Kidney glomerulus stained with anti-Neph (green), CD31 (red), and blood type B antigen (blue). Scale bars indicate 50 µm. **D**, **E** Western blotting analysis using a lysate of sEVs isolated from the plasma of ABO-incompatible kidney transplant recipients, with anti-blood type B antigen and anti-CD9 antibodies. Combinations of donors’ and recipients’ blood types and duration after kidney transplantation are listed above the image. **D** Analysis of several kidney transplant recipients, including negative controls (recipients from non-blood type B donors). **E** Analysis of the same patient whose plasma sEVs were obtained before and after kidney transplantation. *sEVs* small extracellular vesicles, *Pt* patient, *KT* kidney transplantation, *B* blood type B antigen, *Neph* nephrin

To investigate whether kidney-derived sEVs can be identified in peripheral blood, we analyzed plasma sEVs from patients who underwent ABO-incompatible kidney transplants, utilizing the presence of donor blood type antigens as markers for kidney origin. In such situations, sEVs carrying the type B antigens in the peripheral blood of type A recipients were considered to originate from the donor kidney (Fig. 4A). First, we examined the presence of ABO antigens in renal biopsy specimens acquired from kidney transplant recipients. In transplanted kidneys from blood type A recipients who received kidneys from blood type B donors, type B antigens were positive in glomerular endothelial cells, while type A antigens were observed in circulating red blood cells (Fig S2A). Contrarily, both antigens were negative in the renal parenchyma of blood type A recipients who received kidneys from blood type O donors (Fig S2B). In immunofluorescent staining, type B antigens were co-stained with CD31, an endothelial cell marker, in the peritubular capillary and the glomerular endothelial cells (Fig. 4B, C). These results indicated that the ABO blood type antigens in donor kidneys were maintained on the kidney endothelial cells after kidney transplantation.

Subsequently, after confirming that blood type B antigens are present on sEVs of HC, using immunoprecipitation with anti-blood type B antigen antibody-conjugated magnetic beads followed by WB analysis (Fig S3A, B), we isolated sEVs from the plasma of non-blood type B recipients who had received kidneys from blood type B donors. We then examined whether blood type B antigens were detected in the protein assay. WB analysis of sEVs derived from three non-blood type B recipients who received blood type B kidneys revealed the appearance of multiple bands of 75–250 kDa (Fig. 4D). This result was consistent with the finding of a previous study, which reported that blood antigens derived from kidney lysate were present as multiple proteins within 60–270 kDa on WB analysis [28]. Furthermore, these bands appeared soon after kidney transplantation when the donor blood type was B and the recipient blood type was O (Fig. 4E). In summary, WB analysis showed that kidney-derived ABO blood type antigens were present at detectable levels in sEVs from the peripheral blood.

### Kidney-derived sEVs and kidney function in transplant recipients

**Fig. 5:**
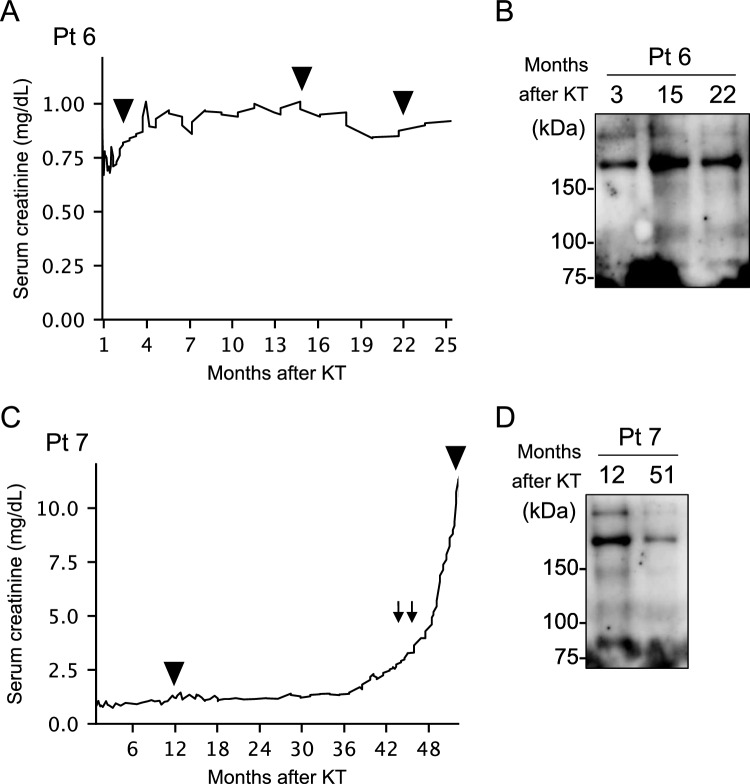
Kidney-derived sEVs and clinical cases of kidney transplant recipients. **A**, **C** The course of kidney function of patients after kidney transplantation. Arrowheads indicate the timing of plasma collection. Arrows indicate the pulse therapy of intra-venous steroids. **B**, **D** Western blotting analysis with anti-blood type B antigen antibodies with the lysate of plasma-derived sEVs from ABO-incompatible kidney transplant recipients. *sEVs* small extracellular vesicles, *Pt* patient, *KT* kidney transplantation

Given the potential of plasma kidney-derived sEVs to mirror the state of a transplanted kidney, we analyzed sEVs possessing the blood type B antigen over time. Patient 6 underwent kidney transplantation due to gout nephropathy and demonstrated preserved graft kidney function (Fig. 5A). In WB analysis, the bands representing the antigens of the transplanted kidney showed stable patterns (Fig. 5B). Conversely, patient 7, who had end-stage renal disease resulting from IgA nephropathy, experienced graft loss after transplantation due to a recurrence of IgA nephropathy (Fig. 5C). Although WB is semi-quantitative, the bands in plasma-derived sEVs, representing their graft antigens, were diminished with the loss of transplanted kidney function (Fig. 5D).

## Discussion

In this study, we gained new insights into kidney-derived sEVs in the plasma. First, we demonstrated that we could detect the signals of plasma-derived sEVs containing mesangial cell marker, ITGA8. Second, by examining sEVs from patients who underwent ABO-incompatible kidney transplantation, we confirmed that the plasma contains detectable levels of sEVs originating from the kidney with the potential for biomarker use.

Mesangial or glomerular endothelial cells are separated from the urinary space by a tight basement membrane (pore size: 2.5–2.8 nm) [8]. Reportedly, sEVs in systemic circulation enter the urine [13]. Theoretically, under physiological conditions, sEVs with a diameter of 50–140 nm may have difficulty passing through the glomerular basement membrane into the urine [8, 15].

Thus, we believed that kidney-derived sEVs from the peripheral blood could be an alternative approach for kidney research. Since almost all types of cells are thought to produce sEVs and release them into circulation [29], developing a method to identify kidney-derived sEVs in blood is of great importance. For cancer-derived EVs, epithelial cell surface molecules including EPCAM were effective markers of pancreatic cancer [30], while endothelial cell-derived EVs with CD31 and CD146 represented atherosclerotic disease markers [31]. To detect sEVs of renal origin, we focused on mesangial cells and one of their surface markers, ITGA8, a candidate marker for mesangial sEVs. ITGA8 expression was observed in vascular smooth muscle cells, alveolar myofibroblasts, and renal mesangial cells [26]. Although ITGA8 expression is not limited to mesangial cells, the above studies indicated that focusing on a subset of EVs might improve the utility. In this study, ITGA8 was expressed both in mesangial cells on renal biopsy and in sEVs from cultured human mesangial cells (Fig. 2B, C, G, and Fig S1). We detected ITGA8 in sEVs from plasma using on-bead flow cytometry. We speculate that some ITGA8-positive plasma-derived sEVs came from kidney mesangial cells and could be utilized for liquid biopsy to detect kidney diseases.

Subsequently, by examining plasma sEVs from patients who underwent ABO-incompatible kidney transplantation, we demonstrated that the renal tissues produced detectable levels of sEVs in the circulation. Compared to that by cancer cells, general cells release fewer EVs into circulation [30]. We examined blood samples from patients who underwent ABO-incompatible kidney transplantation to confirm whether kidney-derived sEVs were detectable in the peripheral blood. We identified sEVs carrying donor blood type antigens, indicating their origin from transplanted kidneys. The patient with a history of kidney injury appeared to have diminished sEVs containing donor blood type antigens. This result is consistent with the previous findings, which employed donor-specific human leukocyte antigens (HLAs) to detect kidney-specific EVs in kidney transplant recipients [31, 32]. While previous studies have employed techniques such as fluorescent nanoparticle detectors or flow cytometry for nano-sized particles [28, 29], our study has demonstrated the possibility of using WB, which can be conducted in any laboratory setting. Further, compared to HLAs, blood type antigens are primarily expressed on endothelial cells (Fig. 4B, C). This could potentially lead to the different property of sEVs with blood type antigens as biomarkers compared to that in HLA-EVs. The reason for the observed decrease in donor-derived sEVs in patients with renal impairment remains unclear. Reportedly, injured transplanted tissues gradually get replaced by recipient-derived cells, leading to tissue chimerism [33]. This progressive decline in donor-derived cells could be one of the explanations for the diminished donor-derived signals.

There are some limitations to this study. First, the sample size was small. Second, investigations of ABO-incompatible kidney transplantation were performed only for blood type B. In our preliminary investigation, the anti-type B antibody displayed greater sensitivity and specificity than did anti-A antibodies; hence, the study participants were limited to recipients who received the kidney from blood type B donors.

In conclusion, we demonstrated the method of detecting sEVs with antigens related to mesangial cells and that plasma contains sEVs originating from the kidney in sufficient amounts for downstream analysis. Our study adds important information to the existing literature on sEVs and their role in disease diagnosis and treatment.

### Supplementary Information

Below is the link to the electronic supplementary material.

Supplementary file1 (DOCX 854 kb)

## Data Availability

The datasets generated and analyzed during the current study are available from the corresponding author upon reasonable request.
