# Supplementary material for: Detecting and exploring kidney-derived extracellular vesicles in plasma
Source: Clin Exp Nephrol. 2024 Mar 4;28(7):617–28. doi: 10.1007/s10157-024-02464-z (PMC11190017; doi:10.1007/s10157-024-02464-z)
Supplement: Supplementary file 1 — Supplementary file1 (DOCX 854 kb) [file 10157_2024_2464_MOESM1_ESM.docx]

**Detecting and exploring kidney-derived extracellular vesicles in plasma**

Shintaro Komatsu^1, 2, 3^, Noritoshi Kato^1*^, Hiroki Kitai^1, 3^, Yoshio Funahashi^1^, Yuhei Noda^1, 3^, Shoma Tsubota^3^, Akihito Tanaka^1^, Yuka Sato^1^, Kayaho Maeda^1^, Shoji Saito^1^, Kazuhiro Furuhashi^1^, Takuji Ishimoto^1^, Tomoki Kosugi^1^, Shoichi Maruyama^1^, Kenji Kadomatsu^3^

^1^Department of Nephrology, Nagoya University Graduate School of Medicine, Nagoya, Aichi, Japan

^2^Division of Molecular Oncology, Center for Neurological Diseases and Cancer, Nagoya University Graduate School of Medicine, Nagoya, Aichi, Japan

^3^Department of Biochemistry, Nagoya University Graduate School of Medicine, Nagoya, Aichi, Japan

^*^ **Corresponding author**:

Noritoshi Kato

Department of Nephrology, Nagoya University Graduate School of Medicine

65 Tsurumai-cho, Showa-ku, Nagoya, Aichi 466-8550, Japan

Tel: +81-52-744-2192 Fax: +81-52-744-2785

Email: n-kato@med.nagoya-u.ac.jp (NK)

**
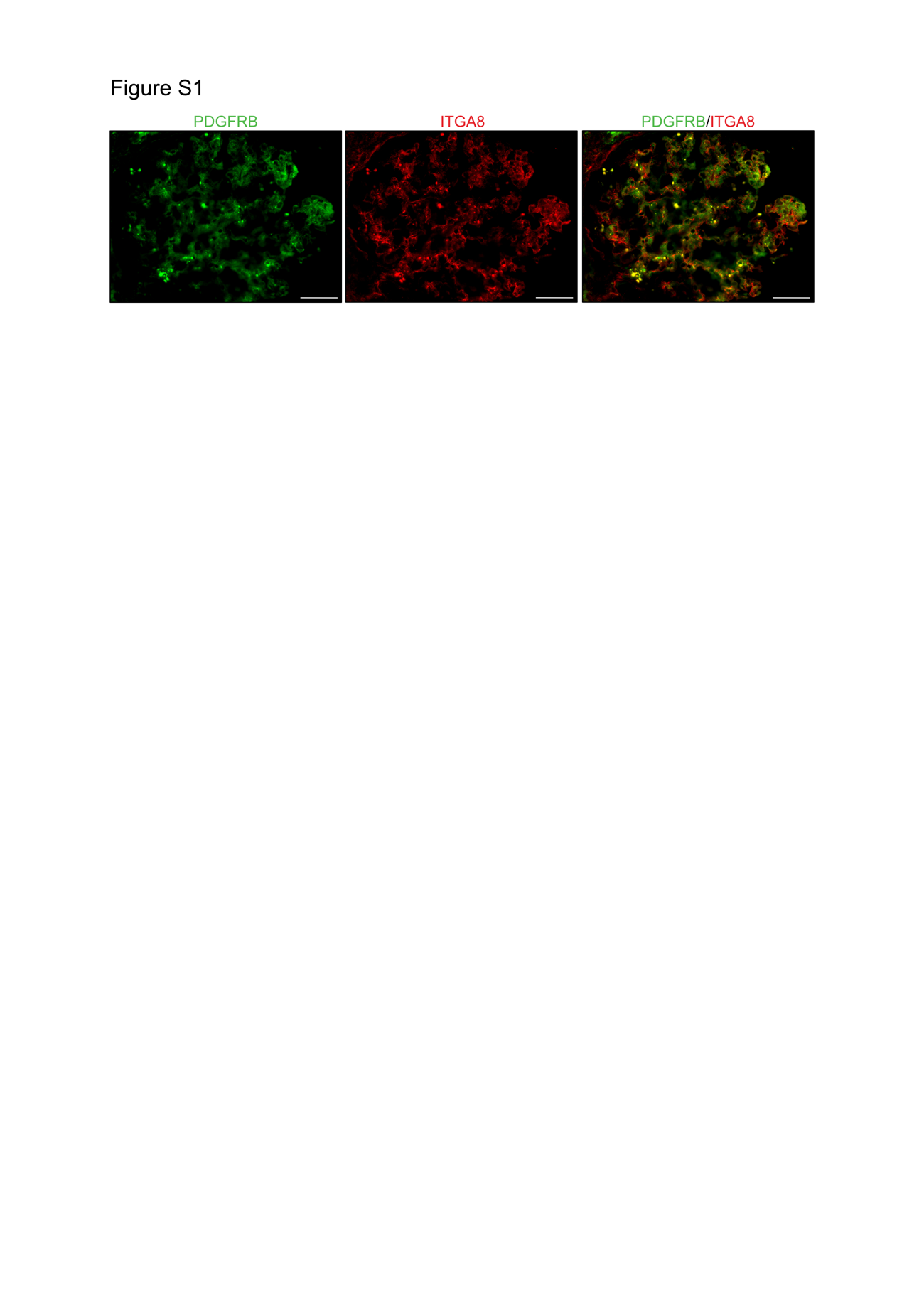
**

**Fig. S1 Immunofluorescent images of human glomeruli**

Immunofluorescent images of human glomeruli stained with PDGFRB (green) and ITGA8 (red). Scale bars indicate 50 µm. *PDGFRB* platelet-derived growth factor receptor Β, *ITGA8* α8 integrin


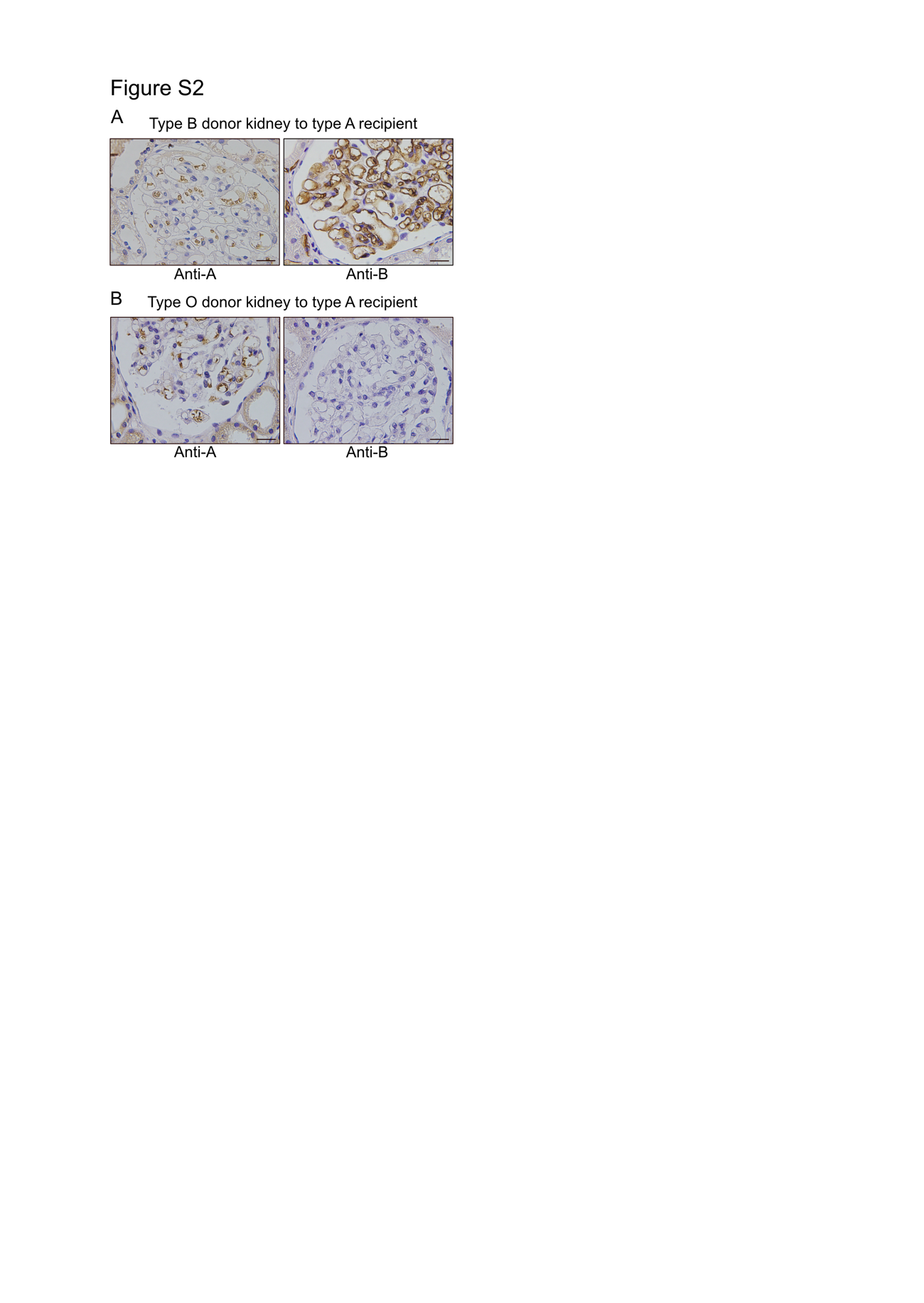


**Fig. S2 Immunohistochemistry of renal biopsy specimens from ABO-incompatible patients with kidney transplants**

**A, B** Immunohistochemistry of renal biopsy specimens stained with anti-blood type A and B antigen antibodies. (A) Donor blood type B kidney to a type A recipient. (B) Donor blood type O kidney to a type A recipient. Scale bars indicate 20 µm.


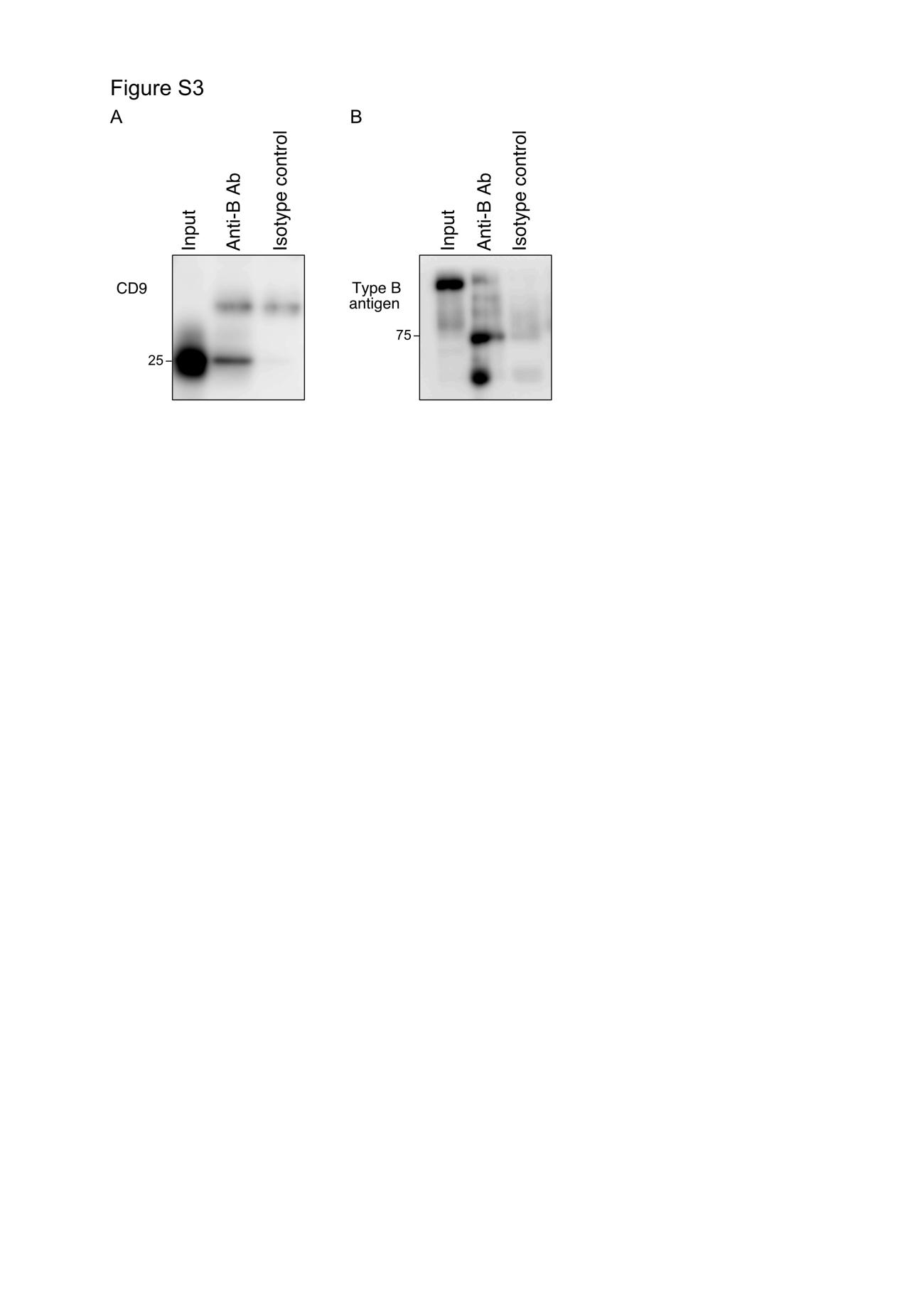


**Fig. S3 Immunoprecipitation using magnetic beads conjugated with anti-blood type B antigen antibody**

Western blotting was performed for anti-CD9 (A) and blood type B antigen (B) using protein lysates of sEVs. These sEVs were derived from the input and captured with anti-blood type B antigen antibody and its isotype control antibody.

*Ab* antibody, *EV* extracellular vesicles
